# The Auditory-Verbal Therapy in Brazil

**DOI:** 10.1590/2317-1782/e20240286en

**Published:** 2025-11-28

**Authors:** Mariane Perin da Silva Comerlatto

**Affiliations:** 1 Universidade Federal de Sergipe – UFS - Lagarto (SE), Brasil.

Dear Editors of CoDAS Journal,

I recently read the newly released book in Brazil titled “Auditory-Verbal Therapy”^(1)^ and would like to recommend it to your readers, along with a few brief comments. This is the first book about Auditory-Verbal Therapy (AVT) written in Portuguese, and it was published by Thieme Revinter in August 2024. The authors offer a thorough and up-to-date exploration of the science, practice, and outcomes of AVT.

The book on Auditory-Verbal Therapy (AVT) is structured into five chapters. The first chapter provides an in-depth analysis of the foundations and principles of AVT, highlighting the importance of collaborative work and detailed therapeutic planning. It also presents a fascinating comparison of different therapeutic approaches based on “Listening and Spoken Language.” The second chapter delves into the historical evolution of AVT and underscores the significance of evidence-based practice. This chapter encourages readers to strive to “be and apply” AVT, a theme that is further developed in the next chapter. The third chapter provides a step-by-step guide for professionals seeking certification and practice in AVT. The fourth chapter presents AVT strategies in a clear and engaging way, offering practical application suggestions and emphasizing the critical role of parent coaching for effective and consistent implementation of therapeutic techniques. Finally, the last chapter shares personal stories from the key protagonists - patients and families - concluding the book with the intention of inspiring transformation in lives.

The foreword deserves special attention, as it energizes the reading experience by immediately introducing the renowned Warren Estabrooks. The author writes:

It is the work of speech-language pathologists, teachers, audiologists, and the entire hearing health care team to guide, navigate, coach and counsel parents on their valuable search for the treasure chest of listening and spoken communication. The professionals in the field help them discover hearing, listening and conversational competence that become their most valued jewels. In AVT parents and professionals form partnerships of hope and trust as they polish these precious gems until they sparkle and dance with life. Professionals continue to capture the child’s attention and the parents’ imaginations as they raise the bar on the global standards in AVT. This is a moment of great opportunity for those who may be interest holders in AVT in the Portuguese speaking world.

In the words of Warren, along with subsequent reflections from Maria Emília, Pedro, and Mariana, we can recognize the transformative power of AVT for children with hearing loss and their families. The question posed at the end of the foreword - *“Why should we accept limitations when so much more is possible?” -* encourages readers to deepen their technical knowledge and pursue new opportunities through AVT certification.

The positive impact of early diagnosis and intervention for hearing loss on the spoken language development of infants and young children is well-established by scientific evidence. Research demonstrates that effective intervention requires a coordinated combination of hearing technologies and specialized therapeutic support. This book affirms and supports the use of AVT as a therapeutic approach grounded in “Listening and Spoken Language.”

In Brazil, several “Listening and Spoken Language” approaches are currently in practice. In recent years, AVT has gained traction, with its certification framework and adherence to the ten fundamental principles increasingly implemented with rigor.

AVT is an internationally recognized certification established in 1978, building practices and research from the 19th century and supported by technological advancements in the 20th-century. Renowned researchers such as Daniel Ling, Helen Beebe, and Doreen Pollack have contributed to structuring the practice. Today, this certification is regulated by the AG Bell Academy and requires, among other criteria, at least 900 hours of clinical practice, specific continuing education, and approximately three to five years of study for completion.

AVT is a dynamic and evolving approach that closely aligns with technological advances and the scientific understanding of typical developmental milestones. It is considered a family-centered early intervention with proven efficacy in developing the listening and spoken language skills of children with hearing loss, whether or not they have associated conditions. These foundational pillars are thoroughly explored and discussed in the pages of the book *Auditory-Verbal Therapy*.

As of August 2025, Brazil has two *Listening and Spoken Language Specialist Certified Auditory-Verbal Therapist* (LSLS Cert. AVT^®^) – one based in São Paulo and the other in Sergipe. Additionally, four professionals are currently working towards: certification (three in São Paulo, and one in Rio Grande do Sul). There are also two certified professionals fluent in Portuguese who live in Canada and Portugal.

While the community of Portuguese-speaking AVT professionals is small, it is strong and has been actively and diligently promoting the practice in Brazil and other Portuguese-speaking nations. A notable achievement from this effort is the publication of the book *Auditory-Verbal Therapy*.

I invite all readers to explore this groundbreaking publication, which, despite being a first in Brazil, offers profound reflections and references from a scientifically validated practice that fulfills the aspirations of countless families worldwide: enabling communication and improving the quality of life of children with hearing loss.
