# Supplementary material for: The Auditory-Verbal Therapy in Brazil
Source: Codas. 2025 Nov 28;37(6):e20240286. doi: 10.1590/2317-1782/e20240286en (PMC12700564; doi:10.1590/2317-1782/e20240286en)
Supplement: Translation

# Translation: A Terapia Auditiva-Verbal no Brasil

**DOI:** 10.1590/2317-1782/e20240286pt

**Authors:** Mariane Perin da Silva Comerlatto

**Affiliations:** 1 Universidade Federal de Sergipe – UFS - Lagarto (SE), Brasil.

Prezados editores da Revista CoDAS,

Li atentamente ao livro lançado recentemente no Brasil “Terapia Auditiva-Verbal”^(1)^ e gostaria de recomendar a leitura e tecer alguns breves comentários para os leitores da Revista CoDAS. O livro é o primeiro escrito em português e foi lançado pela editora Thieme Revinter em agosto de 2024. Nele os autores exploram de forma abrangente e atualizada a ciência, a prática e os resultados da Terapia Auditiva-Verbal (TAV).

As páginas do livro “Terapia Auditiva-Verbal” discorrem cinco capítulos. O primeiro faz uma análise aprofundada e explicita ao leitor os fundamentos e os princípios da TAV, sugere claramente a importância do trabalho colaborativo e da estruturação detalhada de um planejamento terapêutico. Ainda, faz uma curiosa análise da diferença das diferentes abordagens terapêuticas pautadas na “Audição e Linguagem Falada”. O segundo justifica o percurso histórico da TAV e confirma para o leitor o poder de termos uma prática baseada nas evidências científicas. Esse capítulo desperta no leitor o desejo de querer “ser e aplicar” a TAV; anseio que é respondido no capítulo seguinte. Então, o terceiro capítulo destaca o passo a passo para os profissionais que buscam a certificação e a atuação no método. No quarto, de maneira encantadora, são apresentadas as estratégias da TAV com sugestões técnicas de aplicação, que entrelaçam o impacto e a importância do *coaching* de pais para uma prática efetiva e rotineira das técnicas terapêuticas. O último capítulo traz relatos dos protagonistas – os pacientes e familiares – e nele finalizamos o livro com o desejo de querer transformar histórias.

O prefácio merece um destaque especial, pois ele empolga a leitura do livro ao imediatamente reconhecermos o nome de *Warren Estabrooks*. O autor escreve:

É o trabalho de fonoaudiólogos, professores e de toda a equipe de saúde auditiva orientar, navegar, coach e aconselhar os pais em sua incessante busca pelo tesouro da escuta e da comunicação oral. Os profissionais da área devem ajudá-los a descobrir o valor da audição, da escuta e da habilidade conversacional. Na TAV, pais e profissionais formam parcerias em que a esperança e a confiança são fatores fundamentais para lapidar essas preciosas gemas até que elas brilhem e dancem com a vida. Os profissionais continuam a capturar a atenção da criança e a imaginação dos pais à medida que elevam o nível dos padrões globais em TAV. Este é um momento de grande oportunidade para aqueles que se interessam pela TAV no mundo da língua portuguesa.

Nas palavras de Warren – que se somam às reflexões posteriores de Maria Emília, Pedro e Mariana – é possível vislumbrarmos o poder da TAV na transformação das vidas de crianças com deficiência auditiva e suas famílias. A pergunta feita por ele no final do prefácio *–* “*Por que deveríamos aceitar limitações quando tanto mais é possível?” –* empolga o leitor para querer aprofundar sua técnica ao ler o livro e a buscar novos horizontes com a certificação na TAV.

O impacto positivo do diagnóstico e da intervenção precoces das perdas auditivas para o desenvolvimento da linguagem oral de bebês e crianças pequenas é um assunto consolidado pela ciência. As evidências científicas apontam que uma intervenção efetiva é a combinação orquestrada das tecnologias aplicadas à surdez e do acompanhamento terapêutico especializado. E, para tanto, este livro confirma e justifica o uso da TAV como uma abordagem terapêutica baseada na “Audição e Linguagem Falada”.

No Brasil, diferentes métodos pautados na “Audição e Linguagem Falada” estão em prática e, nos últimos anos, a TAV – na sua proposta de certificação e seguindo os exatos 10 princípios que a formam – vem sendo explorada com maior propriedade.

A TAV é uma certificação internacional formalizada em 1978, após práticas e estudos originados no século XIX e endossados pela tecnologia encorpada do século XX. Daniel Ling, Helen Beebe e Doreen Pollack são renomados pesquisadores envolvidos na estruturação da prática. Hoje, o processo de certificação é regulamentado pela *AG Bell Academy* e exige, dentre vários requisitos, ao menos 900 horas de prática clínica, atualizações específicas, e leva em torno de três a cinco anos de estudos para a conclusão.

A TAV é uma abordagem dinâmica e em constante evolução, que acompanha intimamente os avanços da tecnologia e a compreensão científica dos marcos típicos de desenvolvimento. É considerada uma intervenção precoce centrada na família, com eficácia comprovada para o desenvolvimento da audição e da linguagem oral de crianças com deficiência auditiva, com ou sem comprometimentos associados. Tais pilares foram aprofundados e discutidos com propriedade nas páginas do livro “Terapia Auditiva-Verbal”.

Em solo brasileiro, até agosto de 2025, existem duas fonoaudiólogas certificadas em TAV (uma residente em São Paulo e uma em Sergipe) e quatro profissionais em processo de certificação (três em São Paulo e uma no Rio Grande do Sul). Outros dois profissionais certificados e fluentes em língua portuguesa residem no Canadá e em Portugal.

A equipe profissional da TAV em língua portuguesa – apesar de pequena – é sólida e vem trabalhando arduamente para divulgar a prática no Brasil e em outros países de língua portuguesa. O livro “Terapia Auditiva-Verbal” é um dos frutos desse movimento.

Convido a todos a desfrutarem desse lançamento que, apesar de inédito no Brasil, traz referências e reflexões profundas de uma prática cientificamente comprovada que responde aos desejos de inúmeras famílias ao redor do mundo: possibilitar a comunicação e dar qualidade de vida para suas crianças com deficiência auditiva.
